# Artificial intelligence literacy, rather than artificial intelligence anxiety, is associated with career optimism in physiotherapy and rehabilitation students: a cross-sectional study

**DOI:** 10.1186/s12909-026-09899-w

**Published:** 2026-07-09

**Authors:** Mehmet Akif Güler, Senem Demirdel, Ertuğrul Demirdel, Merve Burcu Güler

**Affiliations:** 1 Department of Physiotherapy and Rehabilitation, Faculty of Health Sciences, Selçuk University, Konya, Türkiye; 2 Gülhane Faculty of Physiotherapy and Rehabilitation, Department of Orthopedic Physiotherapy and Rehabilitation, University of Health Sciences, Ankara, Türkiye; 3 Department of Physiotherapy and Rehabilitation, Faculty of Health Sciences, Ankara Yıldırım Beyazıt University, Ankara, Türkiye; 4 Vocational School of Health Services, Elderly Care Program, Selçuk University, Konya, Türkiye

**Keywords:** Artificial intelligence literacy, artificial intelligence anxiety, career optimism, physiotherapy and rehabilitation students, health professions education

## Abstract

**Background:**

Artificial intelligence (AI) is increasingly reshaping health professions education, but its relationship with students’ future career outlook remains insufficiently understood, particularly in professions where human interaction remains central to practice. This study examined whether career optimism in physiotherapy and rehabilitation students was more closely associated with AI literacy than with AI anxiety.

**Methods:**

This cross-sectional study included 438 undergraduate physiotherapy and rehabilitation students from universities in Ankara and Konya, Türkiye. Data were collected online between 27 February 2026 and 10 March 2026 using Turkish versions of the Artificial Intelligence Literacy Scale, Artificial Intelligence Anxiety Scale, and the Career Optimism subscale of the Career Futures Inventory. Pearson correlations, subgroup comparisons, and hierarchical multiple linear regression analyses were performed. HC3 robust standard errors were used in sensitivity analyses.

**Results:**

AI literacy was positively associated with career optimism (*r* = 0.396, *p* < 0.001), whereas AI anxiety showed a weaker negative association (*r* = − 0.147, *p* = 0.002). In hierarchical regression analyses, AI literacy was the only variable independently associated with career optimism in both the crude model (*B* = 0.281, *β* = 0.384, *p* < 0.001) and the adjusted model controlling for age, gender, class year, grade point average, formal AI-related training, and weekly AI use frequency (*B* = 0.284, *β* = 0.388, *p* < 0.001). AI anxiety was not independently associated with career optimism in either model. Sensitivity analyses using HC3 robust standard errors supported these findings.

**Conclusions:**

Among physiotherapy and rehabilitation students, career optimism was more strongly associated with AI literacy than with AI anxiety. Even in a strongly human-centered health profession, students’ career-related expectations appear to be linked more closely to perceived AI-related competence than to AI-related anxiety. Educational strategies may therefore benefit from supporting AI literacy as part of future professional readiness.

**Trial registration:**

Not applicable.

**Supplementary Information:**

The online version contains supplementary material available at https://doi.org/10.1186/s12909-026-09899-w.

## Background

Artificial intelligence (AI) is increasingly becoming part of health care, health professions education, and the broader organization of clinical work. Its applications now extend across clinical decision support, documentation, imaging, risk prediction, remote monitoring, rehabilitation technologies, and educational tools. The JAMA Summit on AI emphasized that AI tools are already being adopted across multiple clinical, operational, and hybrid functions, while also noting that their effects depend strongly on the human–computer interface, user training, implementation context, and ongoing evaluation [1]. In parallel, evidence from medical education shows that AI is being used in areas such as teaching, assessment, admissions, and clinical reasoning, but that ethical guidance, learner preparation, and further research remain important needs [2]. For health professions students, AI is therefore not only a technological innovation but also an emerging component of professional readiness. Future clinicians may be expected not simply to use AI-supported tools, but to understand their capabilities and limitations, evaluate their outputs critically, and integrate them safely into human-centered care [2–5].

This issue is particularly relevant for physiotherapy and rehabilitation. Physiotherapy is a strongly human-centered profession grounded in direct interaction, individualized assessment, clinical reasoning, therapeutic communication, shared decision-making, and continuous adaptation of care according to patient needs [6, 7]. These features make full technological replacement unlikely, especially in clinical situations requiring touch, trust, communication, and individualized therapeutic judgment. At the same time, physiotherapy practice is increasingly influenced by digital and data-supported developments, including telerehabilitation, wearable sensors, movement analysis, remote monitoring, robotics, digital rehabilitation platforms, and AI-supported rehabilitation approaches [8, 9]. Thus, AI may not simply replace physiotherapists, but may alter how physiotherapists assess patients, monitor progress, support adherence, personalize interventions, document care, and communicate clinical information.

Physiotherapy education is also beginning to engage with these changes. Recent evidence suggests that physiotherapy students may view generative AI as useful for learning, clinical reasoning, problem-solving, and individualized study support, while also expressing concerns about learning quality, reliability, permitted use, and the need for a critical approach [10]. In addition, an AI-assisted problem-based learning approach has been reported to improve knowledge retention and AI self-efficacy among undergraduate physiotherapy students, although evidence regarding AI-supported physiotherapy education remains limited [11]. Recent multi-institutional evidence from Türkiye also suggests that physiotherapy students show cautious optimism toward AI-based chatbots, with implications for stage-sensitive integration, AI literacy, ethical awareness, and source verification [12]. These findings indicate that physiotherapy students may encounter AI both as a learning tool and as a possible feature of their future professional environment.

AI literacy and AI anxiety represent two related but conceptually different responses to this changing environment. AI literacy generally refers to the ability to understand, use, evaluate, and reflect on AI systems and their ethical implications [13]. In health professions education, this includes not only general familiarity with AI, but also the ability to recognize the limitations of AI-generated outputs, consider bias and privacy, evaluate reliability, and understand how AI may support rather than replace clinical judgment [2–5, 13]. A recent systematic review of AI literacy scales also showed that most available instruments are self-report measures, with fewer performance-based tools available [14]. This distinction is important because self-reported AI literacy should be interpreted as perceived AI-related competence rather than an objective test of AI knowledge or skill.

AI anxiety, in contrast, refers to feelings of worry, discomfort, or apprehension related to learning about AI, using AI systems, being affected by AI technologies, or facing wider social and occupational consequences of AI implementation [15]. In health professions students, AI anxiety may include concerns about job replacement, technological dependency, reduced human agency, errors, loss of professional autonomy, and uncertainty about future roles. These concerns may be especially complex in physiotherapy because the profession is both human-centered and increasingly exposed to digital transformation. Students may therefore view AI simultaneously as a useful support for learning and practice and as a source of uncertainty about how their future professional role may change.

Career optimism provides a useful outcome for examining these issues. Career optimism refers to positive expectations about future career development and confidence in making appropriate career-related decisions. It is relevant in student populations because career optimism is linked to how individuals interpret future opportunities, cope with career uncertainty, and prepare for professional transitions [16, 17]. In periods of technological change, students’ confidence in their future career may be related not only to traditional academic or clinical preparation, but also to whether they feel capable of understanding and adapting to emerging technologies. For physiotherapy students, AI-related preparedness and AI-related anxiety may therefore be relevant to how they view their future professional development.

Although previous studies have examined AI literacy, AI attitudes, AI readiness, technology acceptance, and AI anxiety in education and health professions contexts, evidence remains limited regarding how these constructs relate to career optimism among physiotherapy and rehabilitation students. This gap is important because physiotherapy represents a profession in which technological change intersects with strongly human-centered clinical practice. Understanding whether career optimism is more closely associated with perceived AI-related competence or with AI-related anxiety may help educators decide whether AI-related educational strategies should focus primarily on reducing fear, strengthening competence, or both. Accordingly, this study examined the associations of AI literacy and AI anxiety with career optimism among undergraduate physiotherapy and rehabilitation students. It was hypothesized that AI literacy would be positively associated with career optimism, whereas AI anxiety would be negatively associated with career optimism.

## Methods

### Study design and setting

This cross-sectional study investigated whether career optimism among undergraduate physiotherapy and rehabilitation students was more strongly associated with artificial intelligence (AI) literacy than with AI anxiety. The study was conducted during the 2025–2026 academic year among students enrolled in Physiotherapy and Rehabilitation programs at universities located in Ankara and Konya, Türkiye.

In Türkiye, undergraduate Physiotherapy and Rehabilitation education is generally delivered as a four-year bachelor’s degree program. The early years of the curriculum typically include foundational biomedical sciences and introductory professional courses, whereas later years include more clinically oriented courses, case-based learning, and supervised clinical practice or internship experiences. Therefore, class year was considered relevant for describing students’ educational stage and potential exposure to clinical and professional learning experiences.

Data were collected through an online questionnaire created in Google Forms between 27 February 2026 and 10 March 2026. The survey link was distributed through student WhatsApp groups that were routinely used for academic communication. The study was reported in accordance with the Strengthening the Reporting of Observational Studies in Epidemiology (STROBE) guideline for cross-sectional studies.

### Participants and eligibility criteria

The target population consisted of undergraduate students enrolled in Physiotherapy and Rehabilitation programs in Ankara and Konya. Participants were recruited using a convenience sampling approach.

Students were eligible if they were enrolled in an undergraduate Physiotherapy and Rehabilitation program at a university in Ankara or Konya, were aged 18 years or older, were able to read and understand Turkish, and agreed to provide electronic informed consent before participation. Only students who provided electronic informed consent and submitted the questionnaire were included in the final dataset.

Students were not eligible for analysis if they were younger than 18 years or were not enrolled in a relevant undergraduate Physiotherapy and Rehabilitation program in Ankara or Konya. Incomplete or unsubmitted questionnaires were not included in the dataset.

### Sample size

Sample size was determined a priori for the planned multiple linear regression analysis. The adjusted model included AI literacy, AI anxiety, age, gender, class year, grade point average (GPA) category, formal AI-related training, and weekly AI use frequency during the past month. After dummy coding of categorical predictors, the adjusted model corresponded to 14 predictor degrees of freedom. Power analysis was performed in R using the pwr package, assuming a small effect size (Cohen’s *f²* = 0.05), a two-sided significance level of *α* = 0.05, and statistical power of 1 − *β* = 0.80. The minimum required sample size was approximately 379 participants. The final analyzed sample consisted of 438 students, exceeding the required sample size.

### Data collection procedure

Participants first viewed an online informed consent page describing the aim of the study, the voluntary nature of participation, and the anonymous handling of responses. Only students who provided electronic consent were able to proceed to the questionnaire. All items in the submitted questionnaire were set as mandatory in Google Forms to minimize item-level missing data. Responses from individuals who exited the survey before final submission were not saved by the platform and were therefore not included in the dataset.

Following data collection, the dataset was screened for potentially invalid or duplicate submissions. This screening included checks for implausibly short response times, repeated demographic-response patterns suggestive of duplicate entries, invariant or near-invariant responses across scale items, and inconsistencies across demographic or academic responses. No directly identifying personal information was collected.

## Measures

### Participant information form

A researcher-developed participant information form was used to collect demographic, academic, and AI-related characteristics. The form included age, gender, class year, university, GPA category, working status, AI use, weekly AI use frequency during the past month, daily AI use duration, academic AI use, and formal AI-related training.

### AI literacy

AI literacy was assessed using the Artificial Intelligence Literacy Scale, originally developed by Wang et al. [18]. The Turkish adaptation study was conducted by Çelebi et al. [19], who reported validity and reliability evidence for use in Turkish-speaking samples. The scale contains 12 items across four subdimensions: awareness, usage, evaluation, and ethics. Each item is rated on a 7-point Likert scale ranging from 1 (strongly disagree) to 7 (strongly agree), with higher scores indicating higher self-reported AI literacy. Negatively worded items were reverse-coded before analysis. Subscale mean scores and a total mean score were calculated.

Because the Artificial Intelligence Literacy Scale is a self-report instrument, scores should be interpreted as perceived AI-related competence rather than as an objective test of AI knowledge or technical skill. In addition, because several subdimensions demonstrated limited internal consistency in the present sample, the primary inferential analyses were based on the total AI literacy score.

### AI anxiety

AI anxiety was assessed using the Artificial Intelligence Anxiety Scale, originally developed by Wang and Wang [15]. The Turkish adaptation study was conducted by Akkaya et al. [20], who reported validity and reliability evidence for the Turkish version. The Turkish version includes 16 items across four subdimensions: learning, job replacement, sociotechnical blindness, and configuration. Items are rated on a 5-point Likert scale ranging from 1 (strongly disagree) to 5 (strongly agree), with higher scores indicating greater AI anxiety. Subscale mean scores and a total mean score were calculated.

### Career optimism

Career optimism was assessed using the Career Optimism subscale of the Career Futures Inventory, originally developed by Rottinghaus et al. [17]. The Turkish adaptation of the Career Futures Inventory was conducted by Kalafat [21], who reported validity and reliability evidence for the Turkish version. The Career Optimism subscale consists of 11 items rated on a 5-point Likert scale ranging from 1 (strongly disagree) to 5 (strongly agree). Higher scores indicate greater career optimism. Reverse-coded items were recoded before scoring, and the mean subscale score was used in all analyses.

### Variables

The primary outcome variable was career optimism. The main predictor variables were AI literacy and AI anxiety. In line with the self-report nature of the Artificial Intelligence Literacy Scale, AI literacy scores were interpreted as perceived AI-related competence rather than objective AI knowledge or technical skill. Age, gender, class year, GPA category, formal AI-related training, and weekly AI use frequency during the past month were included as covariates in the adjusted regression model because of their conceptual relevance to academic progression, academic performance, AI exposure, and future career expectations.

Scale scores were analyzed as continuous variables using mean item scores. Weekly AI use frequency was treated as a grouped categorical variable in subgroup analyses and as an ordinal variable in regression analyses. Gender, class year, and GPA category were dummy coded for regression. Reference categories were female for gender, first year for class year, and GPA ≤ 2.00 for GPA category. In subgroup comparisons by gender, participants selecting “prefer not to say” were excluded because of the very small subgroup size; however, this category was retained in the regression model through dummy coding.

### Data quality and bias reduction

Several measures were used to reduce potential bias and improve data quality. Anonymous online data collection was used to reduce interviewer effects and encourage candid responses. No directly identifying personal information was collected. Mandatory item settings in Google Forms prevented item-level missing data in submitted questionnaires. The dataset was screened after data collection for possible duplicate submissions and invalid response patterns, as described above. Nevertheless, because the study relied on self-reported online data and convenience sampling, recall bias, social desirability bias, and selection bias cannot be completely excluded.

### Use of AI tools

No large language model or generative artificial intelligence tool was used in the design, analysis, interpretation, or writing of this manuscript.

### Statistical analysis

All analyses were performed using R version 4.5.2 (R Foundation for Statistical Computing, Vienna, Austria). Descriptive statistics were used to summarize participant characteristics and scale scores. Internal consistency was evaluated using Cronbach’s *α*.

Associations among AI literacy, AI anxiety, and career optimism were examined using Pearson correlation analysis. Subgroup comparisons were performed using Welch’s independent-samples *t* test or one-way analysis of variance, as appropriate. Effect sizes were reported as Cohen’s *d* for two-group comparisons and eta squared (*η*²) for analysis of variance. Hierarchical multiple linear regression was used to examine the independent associations of AI literacy and AI anxiety with career optimism. In Model 1, AI literacy and AI anxiety were entered simultaneously. In Model 2, age, gender, class year, GPA category, formal AI-related training, and weekly AI use frequency during the past month were added as covariates. Regression results are reported as unstandardized coefficients (*B*), standard errors (SE), standardized beta coefficients (*β*), 95% confidence intervals (CIs), and *p* values. Model diagnostics included assessment of residual patterns, multicollinearity, influential observations, homoscedasticity, and linearity. Because heteroscedasticity was detected in the adjusted model, the model was re-estimated using HC3 heteroscedasticity-robust standard errors as a sensitivity analysis. All tests were two-sided, and statistical significance was set at *p* < 0.05.

## Results

### Participant characteristics

A total of 468 students accessed the online survey form. Of these, 438 provided electronic informed consent and submitted complete questionnaires and were included in the final analysis. The remaining 30 students did not proceed to complete submission. Because the survey invitation was distributed through student WhatsApp groups, the exact number of students who viewed or received the invitation could not be determined. The mean age of participants was 20.5 ± 1.89 years (range, 18–30 years). Most participants were female, and the largest class-year groups were first-year and second-year students. Nearly all participants reported academic use of AI, whereas only a small proportion reported having received formal AI-related training. A summary of the main participant characteristics is presented in Table 1, and additional sample details are provided in Additional file 1: Table S1.

**Table 1:** Participant characteristics

Variable	*n*	%
Gender		
Female	335	76.5
Male	97	22.1
Prefer not to say	6	1.4
Class year		
1st year	145	33.1
2nd year	133	30.4
3rd year	90	20.5
4th year	70	16.0
Grade point average		
≤ 2.00	7	1.6
2.01–2.50	43	9.8
2.51–3.00	162	37.0
3.01–3.50	186	42.5
3.51–4.00	40	9.1
Weekly artificial intelligence use during the past month		
0 days	18	4.1
1–2 days	138	31.5
3–4 days	163	37.2
5–7 days	119	27.2
Daily artificial intelligence use duration		
0–10 min	201	45.9
11–30 min	180	41.1
31–60 min	41	9.4
> 60 min	16	3.7
Academic artificial intelligence use		
No	12	2.7
Yes	426	97.3
Formal artificial intelligence-related training		
No	392	89.5
Yes	46	10.5

Data are presented as n (%) unless otherwise indicated. Mean age = 20.5 ± 1.89 years (range, 18–30 years). Additional sample details, including university and working status, are provided in Additional file 1: Table S1

### Descriptive statistics and internal consistency

Descriptive statistics and internal consistency coefficients for the study measures are presented in Table 2. Internal consistency was acceptable for the total Artificial Intelligence Literacy Scale and the Career Optimism scale, and excellent for the Artificial Intelligence Anxiety Scale. However, several AI literacy subscales, particularly awareness and usage, showed relatively low internal consistency. Therefore, the main inferential analyses were based on the total AI literacy score rather than the subscale scores.

**Table 2:** Descriptive statistics and internal consistency of study measures

Measure/subscale	Items	Mean ± SD	Range	Cronbach’s α
Artificial Intelligence Literacy Scale				
Awareness	3	5.31 ± 1.06	1.67–7.00	0.350
Usage	3	5.26 ± 1.07	1.67–7.00	0.581
Evaluation	3	5.41 ± 1.19	1.00–7.00	0.888
Ethics	3	5.31 ± 1.17	1.00–7.00	0.553
Total	12	5.32 ± 0.92	2.50–7.00	0.849
Artificial Intelligence Anxiety Scale				
Learning	5	2.22 ± 1.01	1.00–5.00	0.951
Job replacement	4	3.08 ± 1.04	1.00–5.00	0.860
Sociotechnical blindness	4	3.19 ± 1.07	1.00–5.00	0.895
Configuration	3	3.03 ± 1.22	1.00–5.00	0.949
Total	16	2.83 ± 0.87	1.00–5.00	0.941
Career Optimism	11	3.46 ± 0.67	1.55–5.00	0.846

Data are presented as mean ± SD and range. *α*, Cronbach’s alpha

### Associations among AI literacy, AI anxiety, and career optimism

**Fig. 1:**
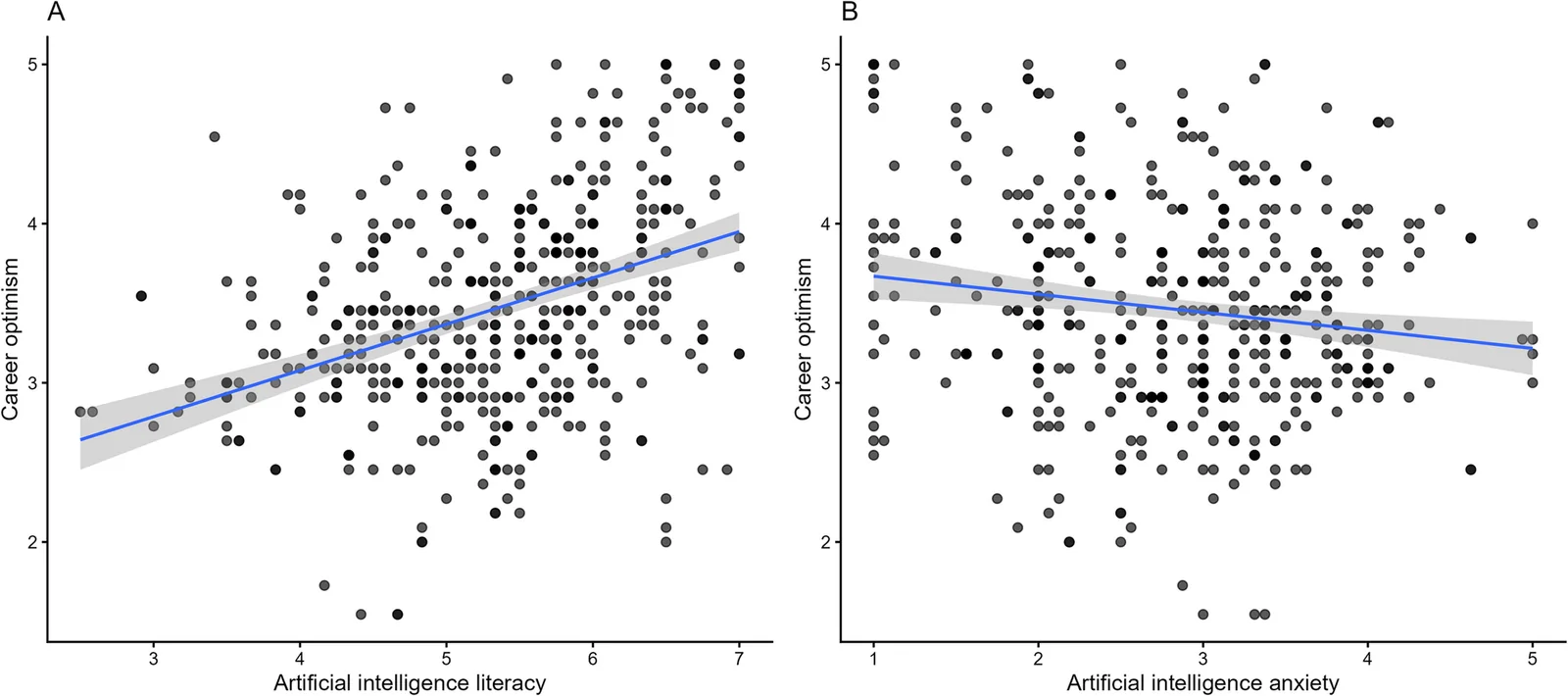
Bivariate associations of career optimism with artificial intelligence literacy and artificial intelligence anxiety. Note. Panel **A** shows the positive association between artificial intelligence literacy and career optimism. Panel **B** shows the negative association between artificial intelligence anxiety and career optimism. Solid lines indicate fitted linear regression lines and shaded areas indicate 95% confidence intervals

Pearson correlation analyses showed that artificial intelligence literacy was moderately and positively associated with career optimism (*r* = 0.396, 95% CI [0.314, 0.472], *p* < 0.001). Artificial intelligence anxiety showed a weaker negative association with career optimism (*r* = − 0.147, 95% CI [− 0.238, − 0.054], *p* = 0.002) and was also negatively associated with artificial intelligence literacy (*r* = − 0.253, 95% CI [− 0.339, − 0.164], *p* < 0.001). Correlation coefficients are presented in Table 3, and the bivariate associations of career optimism with artificial intelligence literacy and artificial intelligence anxiety are shown in Fig. 1.

**Table 3:** Pearson correlations among the main study variables

Variable	1	2	3
1. Artificial intelligence literacy	1		
2. Artificial intelligence anxiety	−0.253***	1	
3. Career optimism	0.396***	−0.147**	1

Values are Pearson correlation coefficients. ** *p* < 0.01; *** *p* < 0.001

### Independent associations with career optimism

Hierarchical multiple linear regression analyses were used to examine the independent associations of AI literacy and AI anxiety with career optimism (Table 4). In Model 1, which included AI literacy and AI anxiety, the model explained 15.9% of the variance in career optimism. AI literacy was positively associated with career optimism, whereas AI anxiety was not independently associated with career optimism.

After adjustment for age, gender, class year, GPA category, formal AI-related training, and weekly AI use frequency in Model 2, AI literacy remained positively associated with career optimism. AI anxiety and the additional covariates were not independently associated with career optimism. The inclusion of covariates increased the explained variance by 2.9%, but this increment was not statistically significant.

Because heteroscedasticity was detected in the adjusted model, HC3 robust standard errors were used in a sensitivity analysis. The results were unchanged: AI literacy remained statistically significant (*B* = 0.284, robust SE = 0.034, 95% CI [0.219, 0.350], *p* < 0.001), whereas AI anxiety remained non-significant (*B* = − 0.040, robust SE = 0.036, 95% CI [− 0.111, 0.031], *p* = 0.268).

**Table 4:** Hierarchical multiple linear regression analysis of career optimism

Variable	Model 1 B (SE)	β	95% CI	*p*	Model 2 B (SE)	β	95% CI	*p*
Intercept	2.074 (0.226)	—	1.630, 2.518	< 0.001	1.544 (0.545)	—	0.473, 2.614	0.005
Artificial intelligence literacy	0.281 (0.033)	0.384	0.216, 0.347	< 0.001	0.284 (0.035)	0.388	0.216, 0.352	< 0.001
Artificial intelligence anxiety	−0.038 (0.035)	−0.050	−0.107, 0.030	0.272	−0.040 (0.036)	−0.052	−0.110, 0.030	0.262
Age	—	—	—	—	0.029 (0.022)	0.081	−0.014, 0.071	0.185
Gender: Male	—	—	—	—	0.033 (0.074)	0.020	−0.113, 0.178	0.661
Gender: Prefer not to say	—	—	—	—	0.114 (0.258)	0.020	−0.394, 0.621	0.659
Class year: 2nd	—	—	—	—	0.081 (0.079)	0.055	−0.075, 0.237	0.307
Class year: 3rd	—	—	—	—	−0.126 (0.101)	−0.076	−0.325, 0.073	0.215
Class year: 4th	—	—	—	—	−0.034 (0.118)	−0.019	−0.267, 0.198	0.773
GPA: 2.01–2.50	—	—	—	—	−0.127 (0.254)	—	−0.626, 0.373	0.618
GPA: 2.51–3.00	—	—	—	—	−0.054 (0.242)	—	−0.530, 0.423	0.824
GPA: 3.01–3.50	—	—	—	—	−0.089 (0.243)	—	−0.566, 0.388	0.715
GPA: 3.51–4.00	—	—	—	—	0.202 (0.257)	—	−0.303, 0.708	0.432
Formal artificial intelligence-related training: Yes	—	—	—	—	0.083 (0.098)	0.038	−0.109, 0.275	0.394
Weekly artificial intelligence use frequency	—	—	—	—	−0.010 (0.017)	−0.027	−0.042, 0.023	0.562

Model 1: *R*² = 0.159, adjusted *R*² = 0.156, *F*(2, 435) = 41.24, *p* < 0.001

Model 2: *R*² = 0.189, adjusted *R*² = 0.162, *F*(14, 423) = 7.02, *p* < 0.001

Δ*R*² = 0.029, Δ*F*(12, 423) = 1.27, *p* = 0.233

*B* = unstandardized coefficient; SE = standard error; *β* = standardized beta coefficient; CI = confidence interval; *R*² = coefficient of determination. Reference categories were female for gender, first year for class year, and ≤ 2.00 for grade point average category. Standardized beta coefficients are not presented for GPA dummy variables

Detailed subgroup comparisons are presented in Additional file 1: Tables S2–S5. Diagnostic results for the adjusted regression model are summarized in Additional file 1: Table S6, HC3 robust regression results are presented in Additional file 1: Table S7, and the corresponding diagnostic plots are shown in Additional file 1: Figs. S1–S3.

## Discussion

This study examined career optimism among physiotherapy and rehabilitation students in relation to self-reported AI literacy and AI anxiety. The principal finding was that AI literacy showed a moderate positive association with career optimism and remained independently associated with career optimism after adjustment for demographic and educational covariates. In contrast, AI anxiety showed a weak negative correlation with career optimism but was not independently associated with career optimism when AI literacy was entered in the same model, either before or after covariate adjustment. These findings should be interpreted as associations rather than causal effects, but they indicate that career optimism in this sample was more closely related to perceived AI-related competence than to AI-related anxiety.

The positive association between AI literacy and career optimism is an important finding in the context of health professions education. Students who perceive themselves as more capable of understanding, using, evaluating, and reflecting on AI systems may also feel more prepared for professional environments affected by technological change. This interpretation is consistent with recent discussions emphasizing that future health professionals will need not only general awareness of AI, but also the ability to evaluate AI outputs critically, understand limitations, consider bias and privacy, and use AI in ways that support safe and human-centered care [2–5, 13]. However, this interpretation requires caution because the AI literacy measure used in this study is a self-report instrument. Therefore, the findings refer to perceived AI-related competence rather than objectively tested AI knowledge or technical skill [14].

The physiotherapy and rehabilitation context is particularly relevant for interpreting these findings. Physiotherapy remains grounded in clinical reasoning, therapeutic communication, individualized assessment, shared decision-making, and direct human interaction [6, 7]. These characteristics make simple technological substitution unlikely. Nevertheless, physiotherapy practice is increasingly influenced by telerehabilitation, wearable sensors, movement analysis, remote monitoring, digital rehabilitation platforms, robotics, and AI-supported rehabilitation technologies [8, 9]. In this context, students’ career optimism may be related to whether they view AI as an understandable and manageable component of future practice rather than as an unclear or uncontrollable source of professional uncertainty.

Recent evidence from physiotherapy education supports this interpretation. Qualitative evidence suggests that physiotherapy students may see generative AI as useful for learning, clinical reasoning, problem-solving, and individualized study support, while also emphasizing the need for critical use, reliability checks, and organizational guidance [10]. Experimental evidence also suggests that structured AI-assisted problem-based learning may improve knowledge retention and AI self-efficacy among physiotherapy students, although evidence in this area remains limited [11]. In Türkiye, recent multi-institutional evidence indicates that physiotherapy students show cautious optimism toward AI-based chatbots, with implications for stage-sensitive integration, ethical awareness, source verification, and AI literacy [12]. The present study extends this emerging literature by focusing not on AI acceptance alone, but on how perceived AI-related competence and AI-related anxiety are associated with students’ career optimism.

The weak negative correlation between AI anxiety and career optimism is also noteworthy. The AI anxiety scale includes concerns related to learning AI, job replacement, sociotechnical blindness, and configuration or use-related difficulties [15]. Such concerns are understandable in a rapidly changing health care environment, particularly when students are uncertain about how AI may affect future professional roles. However, AI anxiety was not independently associated with career optimism when AI literacy was included in the same model. This does not mean that AI-related concerns are unimportant. Rather, it suggests that the bivariate relationship between AI anxiety and career optimism may partly overlap with students’ perceived AI-related competence. Because of the cross-sectional design, this study cannot determine whether higher AI literacy reduces anxiety, whether lower anxiety supports AI learning, or whether both are influenced by other educational or personal factors.

From an educational perspective, the findings suggest that preparing physiotherapy students for an AI-influenced professional environment should not be limited to reassurance about technological change. Educational strategies may benefit from helping students understand what AI systems can and cannot do, evaluate AI-generated information critically, recognize risks related to bias, privacy, and accuracy, and consider how AI can support rather than replace clinical reasoning and therapeutic interaction [1–5, 13]. In physiotherapy education, this may require integrating AI literacy into clinically meaningful learning activities, such as case-based reasoning, evidence appraisal, documentation exercises, rehabilitation technology discussions, and ethical decision-making scenarios.

Overall, the findings suggest that in a strongly human-centered health profession, students’ career-related expectations may be linked more closely to perceived competence and preparedness than to fear alone. This distinction is educationally relevant because it points toward a constructive approach: supporting students to understand, evaluate, and appropriately position AI within their future professional roles, while also acknowledging legitimate concerns about reliability, ethics, professional autonomy, and the preservation of human-centered care.

### Limitations

Several limitations should be considered when interpreting the findings. First, the cross-sectional design does not allow causal relationships or temporal ordering to be inferred. Second, all variables were measured using self-report instruments, which may introduce recall bias, social desirability bias, or common-method bias. In particular, the Artificial Intelligence Literacy Scale should be interpreted as a measure of perceived AI-related competence rather than an objective test of AI knowledge or technical skill. Third, although Turkish validity and reliability evidence is available for the instruments used, the psychometric performance of the AI literacy subscales was not uniform in the present sample. Because some subscales showed relatively low internal consistency, the main analyses focused on the total AI literacy score.

Fourth, the study relied on convenience sampling from universities in Ankara and Konya, which may limit the generalizability of the findings to other institutions, regions, countries, or health professions. Fifth, the survey invitation was distributed through student WhatsApp groups, and the exact number of students who viewed or received the invitation could not be determined. Therefore, the participation flow should be interpreted based on students who accessed the online survey form rather than all potentially eligible students. Finally, heteroscedasticity was detected in the adjusted regression model. Although sensitivity analyses using HC3 robust standard errors produced the same substantive conclusions, the regression findings should still be interpreted with appropriate caution.

### Future directions

Future research should examine how AI literacy develops during physiotherapy and rehabilitation education and whether structured curricular interventions are associated with changes in career optimism, AI anxiety, and professional readiness over time. Longitudinal studies would be particularly valuable for clarifying whether increases in perceived or objective AI competence precede changes in career-related expectations. Intervention studies may also help determine whether AI literacy training, ethical guidance, source-verification exercises, or AI-supported clinical reasoning activities can improve students’ confidence while reducing inappropriate reliance on AI tools.

Future studies should also consider using objective or performance-based measures of AI literacy in addition to self-report instruments. This would help distinguish between perceived AI-related competence and demonstrated ability to evaluate or use AI systems appropriately. In addition, replication in other health professions, universities, and national contexts would be valuable for determining whether the present findings are specific to physiotherapy and rehabilitation students in Türkiye or reflect a broader pattern in health professions education.

## Conclusions

Among physiotherapy and rehabilitation students, AI literacy was positively associated with career optimism and remained independently associated with career optimism after adjustment for demographic and educational covariates. AI anxiety showed a weak negative correlation with career optimism but was not independently associated with career optimism when AI literacy was included in the same model. These findings suggest that students’ career-related expectations in a human-centered health profession may be more closely linked to perceived AI-related competence than to AI-related anxiety. Educational approaches aimed at preparing physiotherapy students for an AI-influenced professional environment may therefore benefit from supporting AI literacy, critical evaluation, ethical awareness, and human-centered integration of AI into future practice.

## Supplementary Information

Supplementary Material 1

## Data Availability

The datasets generated and/or analysed during the current study are not publicly available due to participant privacy considerations but are available from the corresponding author on reasonable request.

## Abbreviations

AI: Artificial intelligence
CI: Confidence interval
GPA: Grade point average
HC3: Heteroscedasticity-consistent robust standard error estimator
SD: Standard deviation
SE: Standard error
STROBE: Strengthening the Reporting of Observational Studies in Epidemiology
