# Supplementary material for: Artificial intelligence literacy, rather than artificial intelligence anxiety, is associated with career optimism in physiotherapy and rehabilitation students: a cross-sectional study
Source: BMC Med Educ. 2026 Jul 9;26:1462. doi: 10.1186/s12909-026-09899-w (PMC13563800; doi:10.1186/s12909-026-09899-w)
Supplement: Supplementary file 1 [file 12909_2026_9899_MOESM1_ESM.pdf]

Additional file 1: Supplementary Tables S1–S7 and Supplementary Figures S1–S3.

Supplementary Table S1 Additional participant characteristics

**Variable**	*n*	%
**University**		
Selçuk University	175	39.9
Ankara Yıldırım Beyazıt University	139	31.7
Health Sciences University	104	23.7
Yüksek İhtisas University	14	3.2
Ankara Medipol University	3	0.7
Atılım University	2	0.5
Necmettin Erbakan University	1	0.2
**Working status**		
Not working	381	87.0
Part-time	47	10.7
Full-time	10	2.3

Note. Data are presented as *n* (%).

Supplementary Table S2 Gender-based comparisons of artificial intelligence literacy, artificial intelligence anxiety, and career optimism

**Variable**	Female (*n* = 335) Mean ± SD	Male (*n* = 97) Mean ± SD	*t*	*p*	Cohen's *d*
Artificial intelligence literacy	5.36 ± 0.88	5.23 ± 1.07	1.11	0.267	0.14
Artificial intelligence anxiety	2.86 ± 0.84	2.67 ± 0.96	1.76	0.081	0.22
Career optimism	3.46 ± 0.67	3.47 ± 0.69	-0.09	0.930	-0.01

Note. Participants who selected "prefer not to say" for gender (*n* = 6) were excluded from inferential gender analyses because of the small group size. Welch's *t* test was used.

Supplementary Table S3 Class-year comparisons of artificial intelligence literacy, artificial intelligence anxiety, and career optimism

**Variable**	1st year (*n* = 145)	2nd year (*n* = 133)	3rd year (*n* = 90)	4th year (*n* = 70)	*F*	*p*	η^2
Artificial intelligence literacy	5.24 ± 1.01	5.22 ± 0.86	5.51 ± 0.81	5.45 ± 0.94	2.61	0.051	0.020
Artificial intelligence anxiety	2.82 ± 0.79	2.87 ± 0.85	2.87 ± 0.97	2.73 ± 0.97	0.44	0.728	0.003
Career optimism	3.41 ± 0.68	3.50 ± 0.63	3.44 ± 0.71	3.53 ± 0.69	0.78	0.505	0.005

Note. One-way ANOVA was used. For artificial intelligence literacy, Tukey post hoc comparisons showed no statistically significant pairwise differences.

Supplementary Table S4 Comparisons according to formal artificial intelligence-related training

**Variable**	No (*n* = 392) Mean ± SD	Yes (*n* = 46) Mean ± SD	*t*	*p*	Cohen's *d*
Artificial intelligence literacy	5.28 ± 0.93	5.64 ± 0.79	-2.87	0.006	-0.39
Artificial intelligence anxiety	2.83 ± 0.86	2.81 ± 0.98	0.18	0.858	0.03

Variable	No (n = 392) Mean ± SD	Yes (n = 46) Mean ± SD	t	p	Cohen's d
Career optimism	3.44 ± 0.67	3.61 ± 0.69	-1.55	0.126	-0.25

Note. Welch's t test was used.

Supplementary Table S5 Comparisons according to weekly artificial intelligence use frequency during the past month

Variable	0 days (n = 18)	1–2 days (n = 138)	3–4 days (n = 163)	5–7 days (n = 119)	F	p	η^2
Artificial intelligence literacy	4.60 ± 1.02	5.20 ± 0.84	5.28 ± 0.92	5.64 ± 0.89	9.91	<0.001	0.060
Artificial intelligence anxiety	3.20 ± 0.71	2.97 ± 0.90	2.80 ± 0.79	2.65 ± 0.95	4.12	0.007	0.030
Career optimism	3.46 ± 0.48	3.46 ± 0.61	3.39 ± 0.69	3.57 ± 0.73	1.55	0.201	0.010

Note: One-way ANOVA was used. Tukey post hoc tests showed that artificial intelligence literacy scores were significantly higher in the 1–2 days (p = 0.039), 3–4 days (p = 0.013), and 5–7 days (p < 0.001) groups compared with the 0 days group. In addition, the 5–7 days group scored higher than the 1–2 days (p = 0.001) and 3–4 days (p = 0.004) groups. For artificial intelligence anxiety, only the difference between the 5–7 days and 1–2 days groups was significant (p = 0.017).

Supplementary Table S6 Regression diagnostics for the adjusted model of career optimism

**Diagnostic indicator**	Value
Shapiro–Wilk test for residuals, W	0.995
Shapiro–Wilk test for residuals, p	0.153
Studentized Breusch–Pagan test, BP	31.707
Breusch–Pagan test, df	14
Breusch–Pagan test, p	0.004
Maximum Cook's distance	0.055
4/n threshold	0.009
Number of observations above 4/n threshold	23
Adjusted GVIF range	1.016–1.390

Note: Residuals were approximately normally distributed, and adjusted GVIF values indicated low multicollinearity. Although 23 observations exceeded the conservative 4/n Cook's distance screening threshold, the maximum Cook's distance was 0.055, suggesting that no single observation exerted undue influence on the model. The studentized Breusch–Pagan test suggested heteroscedasticity; therefore, HC3 robust standard errors were used in the sensitivity analysis.

Supplementary Table S7 Hierarchical regression results using HC3 robust standard errors for the adjusted model

**Variable**	Estimate	Robust SE	95% CI	p
Intercept	1.544	0.557	0.453, 2.635	0.006
Artificial intelligence literacy	0.284	0.034	0.219, 0.350	<0.001
Artificial intelligence anxiety	−0.040	0.036	−0.111, 0.031	0.268
Age	0.029	0.020	−0.010, 0.068	0.148

**Variable**	Estimate	Robust SE	95% CI	*p*
Gender: Male	0.033	0.072	−0.109, 0.174	0.652
Gender: Prefer not to say	0.114	0.094	−0.070, 0.298	0.226
Class year: 2nd	0.081	0.079	−0.075, 0.237	0.309
Class year: 3rd	−0.126	0.108	−0.338, 0.086	0.245
Class year: 4th	−0.034	0.121	−0.271, 0.202	0.777
GPA: 2.01–2.50	−0.127	0.348	−0.810, 0.556	0.716
GPA: 2.51–3.00	−0.054	0.342	−0.725, 0.617	0.875
GPA: 3.01–3.50	−0.089	0.341	−0.758, 0.580	0.795
GPA: 3.51–4.00	0.202	0.345	−0.473, 0.878	0.558
Formal artificial intelligence-related training: Yes	0.083	0.096	−0.105, 0.272	0.387
Weekly artificial intelligence use frequency	−0.010	0.017	−0.044, 0.025	0.579

Note. HC3 = heteroscedasticity-consistent robust standard error estimator. Confidence intervals are based on robust standard errors. The robust analysis confirmed the same substantive pattern as the conventional adjusted regression model: artificial intelligence literacy remained the only variable independently associated with career optimism.

Supplementary Fig. S1 Residuals versus fitted values for the adjusted model.

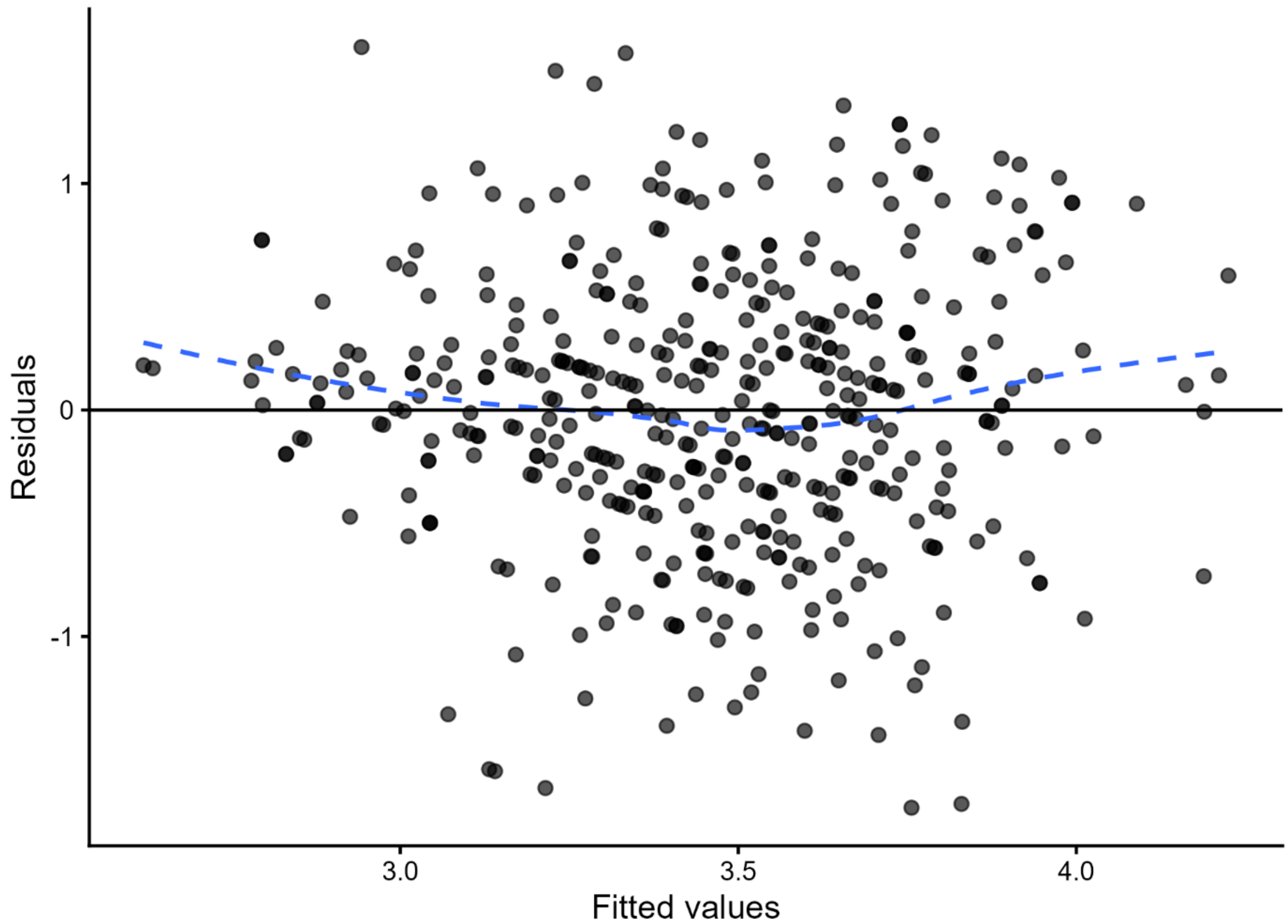

Note. The plot was used for visual assessment of linearity and variance patterns in the adjusted regression model.

Supplementary Fig. S2 Normal Q–Q plot of residuals for the adjusted model.

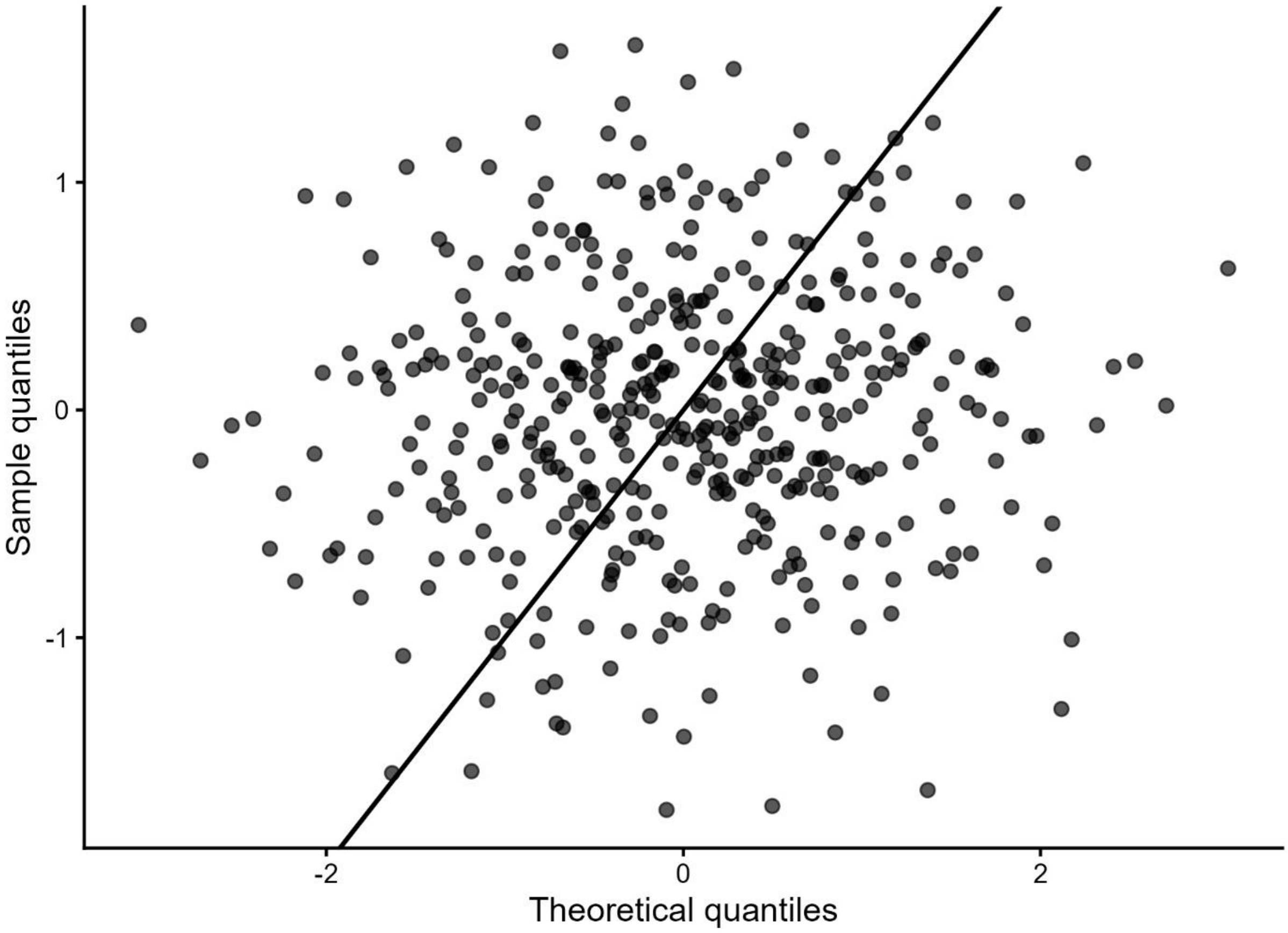

Note. The plot was used for visual assessment of residual normality in the adjusted regression model.

Supplementary Fig. S3 Cook's distance values for the adjusted model.

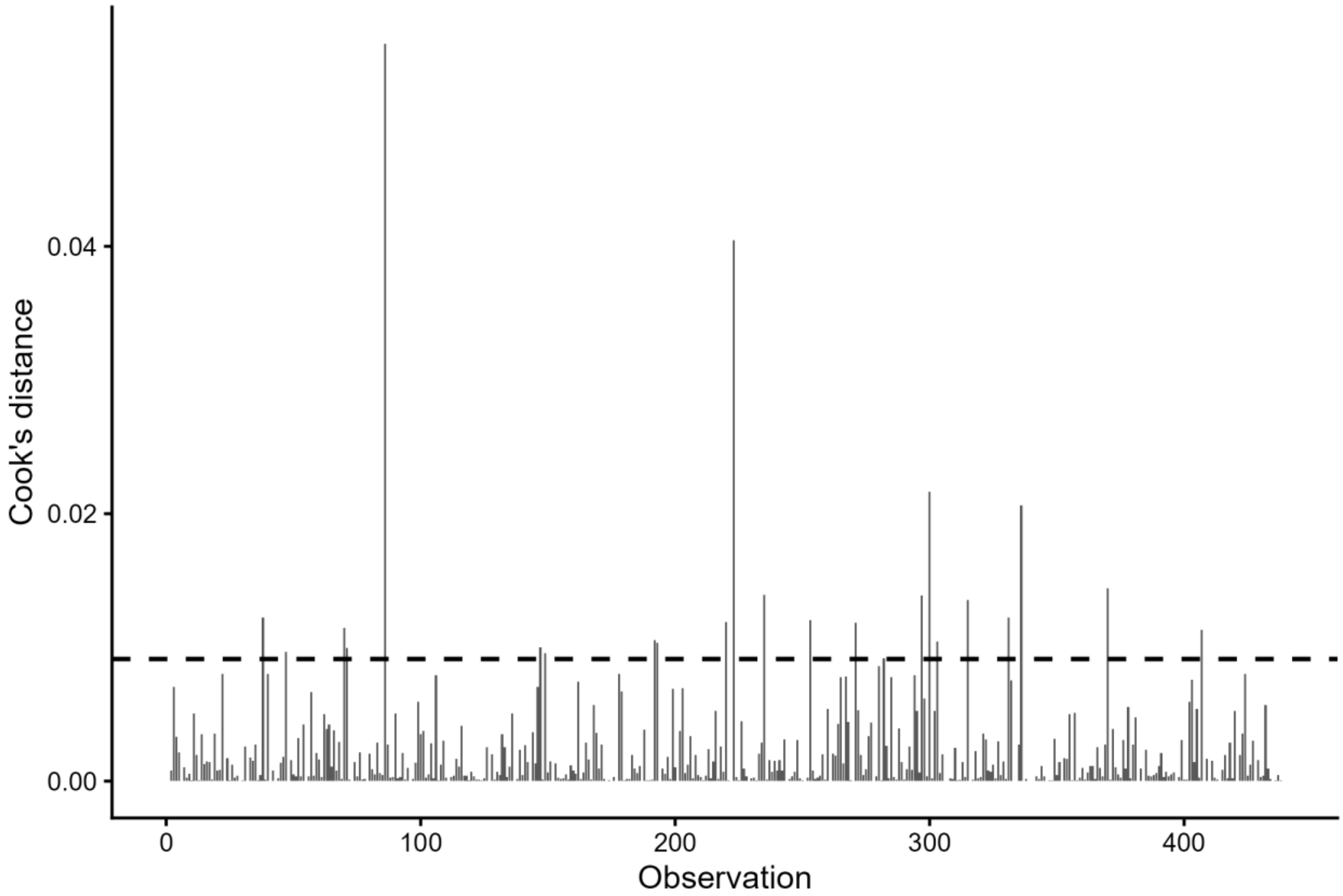

Note. The plot was used to inspect potentially influential observations in the adjusted regression model.
